# I know how you’ll say it: evidence of speaker-specific speech prediction

**DOI:** 10.3758/s13423-024-02488-2

**Published:** 2024-03-25

**Authors:** Marco Sala, Francesco Vespignani, Laura Casalino, Francesca Peressotti

**Affiliations:** 1 Departement of Developmental Psychology and Socialization, University of Padua, Padova, Italy; 2 Padua Neuroscience Center, University of Padua, Padova, Italy

**Keywords:** Foreign accent, Language prediction, Speech prediction

## Abstract

Most models of language comprehension assume that the linguistic system is able to pre-activate phonological information. However, the evidence for phonological prediction is mixed and controversial. In this study, we implement a paradigm that capitalizes on the fact that foreign speakers usually make phonological errors. We investigate whether speaker identity (native vs. foreign) is used to make specific phonological predictions. Fifty-two participants were recruited to read sentence frames followed by a last spoken word which was uttered by either a native or a foreign speaker. They were required to perform a lexical decision on the last spoken word, which could be either semantically predictable or not. Speaker identity (native vs. foreign) may or may not be cued by the face of the speaker. We observed that the face cue is effective in speeding up the lexical decision when the word is predictable, but it is not effective when the word is not predictable. This result shows that speech prediction takes into account the phonological variability between speakers, suggesting that it is possible to pre-activate in a detailed and specific way the phonological representation of a predictable word.

**Supplementary Information:**

The online version contains supplementary material available at 10.3758/s13423-024-02488-2.

## Introduction

Classic models of language comprehension have long recognized the significant role of prediction. Traditionally, it was believed that the parser could predict the part of speech (grammatical category) of forthcoming words in a sentence (Kimball, 1975). Within the garden path model, this information is used to predict the minimal grammatical completion of the fragment read so far (Staub & Clifton, 2006). However, there has been ongoing controversy surrounding whether and how the system also predicts specific words, at least in highly constraining contexts. Initially, skepticism surrounded this notion, as it was considered wasteful to predict incoming information, given the large number of sensible sentence continuations (Forster, 1981; Jackendoff, 2002; see also Van Petten & Luka, 2012). Nevertheless, a large body of empirical evidence has now dispelled this skepticism, embracing the idea that language comprehension involves context-based pre-activation of upcoming words, which facilitates the bottom-up processing of linguistic input (Altmann & Mirković, 2009; Dell & Chang, 2014; Kutas et al., 2011; Pickering & Garrod, 2007, 2013).

Despite the widespread acceptance that people implicitly predict upcoming linguistic information, the underlying processes are not yet fully understood. The debate focuses on the nature of the processes and the representations involved in prediction, as well as the circumstances under which prediction occurs. Traditionally it was assumed that the pre-activation of upcoming words arises from the passive spreading of activation between pre-existing representations, which are (partially) activated during the processing of the context (Anderson, 1983; Collins & Loftus, 1975; Hutchison, 2003; Huettig et al., 2022; McRae et al., 1997). More recent models of language comprehension do not consider spreading of activation as the only mechanism involved and highlight the pro-active aspect of internal prediction generation (Federmeier, 2007; Huettig, 2015; Kuperberg & Jaeger, 2016; Pickering & Gambi, 2018; Pickering & Garrod, 2013). In this context, prediction-by-production models have received particular attention. These models propose that prediction during comprehension can be implemented by using representations and mechanisms that are also employed in language production (Huettig, 2015; Pickering & Gambi, 2018; Pickering & Garrod, 2013).

Another issue in the language prediction literature concerns when and to what extent higher-level internal information can be used to pre-activate upcoming information. It has been proposed that predictive processes come with a cost and may only be implemented when the context is highly constraining and when sufficient resources and time are available (Pickering & Gambi, 2018), or when predictions are particularly useful (Kuperberg & Jaeger, 2016). Additionally, prediction models usually assume that highly constraining contexts allow for the pre-activation of sub-lexical information. However, while there is solid evidence in favor of semantic (Altmann & Kamide, 1999, 2007; Chambers et al., 2002; Federmeier & Kutas, 1999; Kamide et al., 2003; Metusalem et al., 2012; Paczynski & Kuperberg, 2012) and syntactic (Crocker, 2000; Kimball, 1975; Levy, 2008; Lewis, 2000; Staub & Clifton, 2006; Traxler, 2014; Traxler et al., 1998; van Gompel et al., 2005) pre-activation, the evidence for prediction of the phonological form of the upcoming word is not fully consistent (DeLong et al., 2005; Heilbron et al., 2022; Ito et al., 2016, 2017, 2018, 2020; Ito & Sakai, 2021; Martin et al., 2013; Nicenboim et al., 2020; Nieuwland et al., 2018).

The ERP study by DeLong et al. (2005) was the first to provide evidence of phonological prediction. They investigated the modulation of the N400 amplitude elicited by an indefinite article which could agree or disagree with the phonological form of the semantically predictable following noun. The results showed the N400 modulation at the presentation of the article, as a function of its expectancy. This indicates that participants predicted the phonological form of the word, leading to increased negativity when the article mismatches with this prediction. Despite the strong theoretical impact of the DeLong et al. (2005) study, subsequent research has tried to replicate the N400 modulation on the pre-target article with mixed results (Ito et al., 2017, 2020; Martin et al., 2013; Nicenboim et al., 2020; Nieuwland et al., 2018).

Another relevant challenge for phonological prediction is the acoustic variability of speech. Speakers vary in the physical realization of speech sounds (Liberman et al., 1967). This is particularly evident in non-native speech, which deviates not only phonetically but frequently also at the phonological level (Best et al., 2001; Clopper et al., 2005; Flege, 1988). Listeners, however, demonstrate the ability to adapt to non-native speech (Bradlow & Bent, 2008; Clarke & Garrett, 2004; Clopper & Bradlow, 2008; Maye et al., 2008), and imitation of a foreign accent seems to facilitate such perceptual adaptation (Adank et al., 2010).

**Fig. 1:**
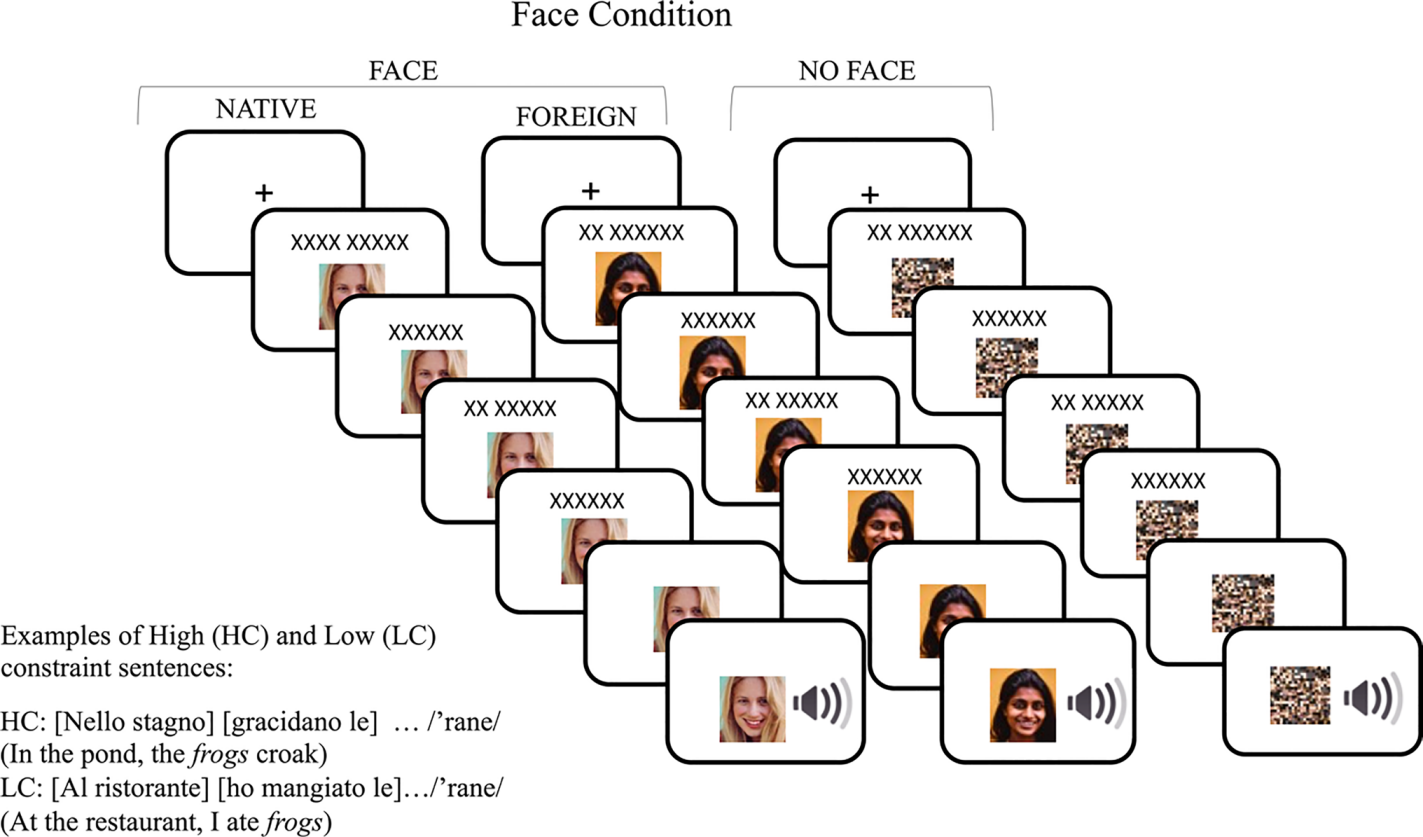
Schematic representation of the experimental paradigm and procedure. *Note.* A trial consisted of a variable number of frames exposed for 800 ms each. The number of frames depended on the length of each sentence. In each frame, a sentence fragment was presented together with a visual stimulus that could be either the face of the native or foreign speaker or a control stimulus. The face cued the accent of the target word (in the example RANE/ frogs) that was presented auditorily, while the speaker’s face or the control stimulus remained visible. Sentences could be highly constraining (HC) or low constraining (LC) towards the target word

The present study aims to provide evidence in favor of prediction at the phonological level by exposing listeners to misspelled target words uttered by a foreign-accented speaker. Participants were asked to read sentence frames in which the final word was uttered by either a native or a foreign speaker. The foreign speaker’s words contained a consistent phonological error in the first phoneme of the word. Before the experiment, participants were familiarized with both native and foreign speakers. The speaker’s face was presented along with a 1-min audio clip in which each speaker introduced themselves. Experimental trials consisted of reading a sentence frame that missed a last word to be grammatical. The last word was presented acoustically, and participants were required to perform an auditory lexical decision task on the spoken word. The last word could be either predictable or not based on the preceding sentential context. A further critical manipulation was that the speaker’s accent could be either predictable or not, as the written sentences were presented in association with the speaker’s face or with a neutral visual stimulus (see Fig. 1).

One key feature of the present paradigm is the choice to present the sentential context in written form. This was driven by the theoretical requirement of dissociating the effect of the prediction of foreign-accented phonology from the possible cognitive load increase associated with processing the sentence context with an unusual (foreign) phonology. Previous studies often confounded these two effects by presenting both the context and the target words with a native or a foreign accent (Brunellière & Soto-Faraco, 2013; Porretta et al., 2020; Romero-Rivas et al., 2016). In contrast, written form presentation ensures that the cognitive load associated with the processing of the context is matched for both the native and the non-native speaker.

The use of the speaker face cue during reading is expected to facilitate the perception and recognition of the target word. Specifically, we hypothesize a facilitatory effect of the face cue in the lexical decision response times only when the context is highly constraining, as this should allow the prediction of the semantic and phonological features of the target word. If the effect is to be attributed to prediction, no effect of face manipulation on response times should emerge in the low-constraint condition. We are also interested in investigating whether the use of information about the speaker’s accent to predict upcoming speech differs between the native (expecting standard phonology) and the foreign accent (expecting a specific deviant phonology).

### Method

#### Participants

Fifty-four adults (40 females, mean_age_ = 23.80 years, SD = 2.97) were recruited. Participants were native Italian speakers with no history of neurological, language-related, or psychiatric disorders. Two participants were excluded from the analyses due to a recruitment error (one person had participated in the stimulus norming cloze probability test, and the other was not a native Italian speaker). The final sample consisted of 52 participants (40 females, mean age = 23.67 years, SD = 2.96). Since no reliable estimate of the effect sizes of our interest was available, the sample size was determined a priori based on the recommendation that regression analyses should include five to ten observations per variable to give an acceptable estimate of regression coefficients (Hair et al., 2010; Tabachnick & Fidell, 1989). It is important to note that the total number of observations in linear mixed-effects models includes both the number of participants and the number of observations nested within each participant per variable (Bates et al., 2015). The research adhered to the principles outlined in the Declaration of Helsinki. Participants provided their informed consent before participating in the experiment. The research protocol was approved by the Ethics Committee for Psychological Research of the University of Padova (protocol number: 5181).

### Materials

The materials are available via the Open Science Framework (OSF) repository of the current project (https://osf.io/n42x9/). The target stimuli consisted of 64 spoken words (mean length = 6.23 phonemes, SD = 1.97) and 64 spoken non-words (mean length = 6.45 phonemes, SD = 1.98) beginning with the phonemes /r/, /p/ and /k/. These three phonemes were never present in any other position of words and non-words. Given that in the foreign accent condition participants were asked to discriminate mispronounced words and non-words, non-words had no phonological neighbors and could be easily identified as such. Each target word was preceded by a written sentential context that could be either High constraining (example in 1a) or Low constraining (example in 1b) toward the target word.

- Nello stagno gracidano le **rane**
  In-the pond croak the **frogs**
  ‘In the pond the **frogs** croak’
- Al ristorante ho mangiato le **rane**
  At-the restaurant have eaten the **frogs**
  ‘At the restaurant I ate **frogs**’

To determine the constraining level, an online sentence completion questionnaire was administered to 22 participants, who did not take part in the experiment. They were instructed to complete each sentence frame with the first word that came to mind. The sentence constraint was operationalized as the proportion of total responses involving the most frequent continuation (HC constraint: mean = 0.94, SD = 0.07; LC constraint: mean = 0.18, SD = 0.08). The target word in High-Constraint sentence frames was the most frequent continuation. The target word in Low-Constraint sentences was always a semantically plausible continuation. The sentence frames varied in length (mean = 9.00 words, SD = 2.00, range = 4–14), but their length was matched between conditions (HC sentences: mean = 9.14 words, SD = 2.12, range = 4–14; LC sentences: mean = 8.86 words, SD = 1.88, range= 4–13; *p* = .429). An equal number of written sentences, matched for length (HC sentences: mean = 9.31, SD = 2.05, range= 5–15; LC sentences: mean = 8.83, SD = 1.90, range= 5–15; *p* = .167) and level of constraint (HC constraint: mean = 0.90, SD = 0.11; LC constraint: mean = 0.22, SD = 0.08) to the sentences preceding words, were used to precede the nonwords.

The speech stimuli were spoken by an artificial voice with a native accent in one condition and a voice with a foreign accent in the other condition. All stimuli of the native and foreign conditions were synthesized using the Microsoft Azure text-to-speech service. Microsoft Azure allows to synthesize speech stimuli using the prebuilt neural voices of speakers of different languages. We used two different speakers for the native and foreign-accented conditions. For the native-accented voice, the prebuilt neural voice of the Italian speaker Fabiola was selected. For the foreign-accented voice, the prebuilt neural voice of the Indian speaker Neerja was selected. The two speakers were selected with the aim of making participants associate a given face stimuli with a foreign/native speaker voice. To control for participant’s familiarity with different foreign accents, the foreign accent was artificially created by modifying three phonemes (/r/, /p/ and /k/) produced by the foreign-accented voice as /l/, /b/ and /ɢ/, respectively. The novel foreign accent was created by manipulating the place or manner of articulation of the target phonemes. This choice was aimed at obtaining a foreign accent that was easily perceived by the listener but at the same time sounded natural. To minimize the impact of the phonetic variability between the native and the foreign voice, all stimuli were synthesized starting from the IPA encoding of the words and non-words in Italian. The phonological manipulation of the foreign-accented speaker was implemented by changing the target phonemes in the IPA encoding of the stimuli (e.g., the target stimulus was /kˈaldo/ for the native speaker voice and /ɢ'aldo/ for the foreign speaker voice). In this way, both the native and the foreign speakers received the same phonetic sequences as input, thus, there were no other phonetic approximations or differences between the voices except for the phonological manipulation. The foreign-accented voice mispronounced the initial phoneme of all target stimuli. The mispronounced words did not correspond to any existing Italian word. The phonological manipulation was implemented to synthesize both the experimental stimuli and the familiarization speech of the foreign speaker. The native speaker voice and the foreign-accented voice differ in prosody, but this was only perceivable in the familiarization phase where participants were exposed to lengthy sections of text.

### Procedure and design

The experiment was carried out using Psychopy (Peirce et al., 2019). Participants were tested in a quiet room while wearing headphones. In the first familiarization phase, participants viewed a picture of the speaker’s face and listened to a one-minute speech in which the speaker introduced herself. Two different speeches of the same length were prepared, each associated with a different face. These speeches were synthesized either with the native-accented voice or with the foreign-accented voice. The computer screen displayed a face, either an Indian-looking female face (for the foreign-accented speaker) or an Italian-looking female face (for the native speaker). Both speeches were presented to participants in two parts of 30 s each, alternating between speakers. Then the experimental stage followed. The participants were instructed to read the sentence frames displayed on the screen and to judge whether the subsequent auditory target was a word or not. The auditory target could be pronounced by the familiarized native speaker or the familiarized foreign-accented speaker. The speaker’s face was presented 2,500 ms before the sentence, 4.5 cm below the center of the screen, and remained visible throughout the whole trial. In half of the trials, the face was replaced by a control stimulus, which was a scrambled version of the faces of the two speakers. Both the face and the control stimulus were 10 cm wide and 10 cm high. The presentation of each sentence started with a fixation point appearing 4.5 cm above the center of the screen for 50 ms. The sentence frames were then presented phrase-by-phrase, with each phrase presented for 800 ms, followed by a 150-ms inter-phrase interval. The auditory target stimuli (word or nonword) were presented 800 ms after the presentation of the last phrase of the sentence. Participants were instructed to categorize the spoken targets as words or non-words by pressing the ‘M’ or ‘C’ keys on the keyboard with their left and right index. They were asked to respond to words with the index finger of their dominant hand. In the case of foreign accent, participants were explicitly asked to accept mispronounced words as real words. Response times were recorded from the presentation of the target stimulus for a maximum time of 2,000 ms after the end of the stimulus. To encourage participants to read the sentences, a comprehension question requiring a forced choice yes / no response was presented in 10% of trials (20% of trials with words as target) after the lexical decision. The participants used the same keys as in the lexical decision, using the finger of the dominant hand to provide the “Yes” response. Before starting the experimental session, participants completed 12 practice trials that were not part of the experimental materials. The session lasted about 45 min.

Each participant was presented with 256 trials, 128 ending with a word and 128 with a non-word. Target words and non-words appeared in High and Low constraining sentence frames. The experimental material was divided into two blocks, ensuring that each word or non-word appeared only once per block. Within each block, 64 speech stimuli (32 words, 32 non-words) were spoken by the native speaker and 64 by the foreign speaker. The assignment of speech stimuli to the native or foreign speaker was counterbalanced between blocks. The speaker’s accent was either cued or not cued by the speaker’s face, resulting in 16 observations per cell. The order of the two blocks was counterbalanced between participants and the order of the trials within blocks was randomized. To ensure that each stimulus was presented in both native and foreign accents, and with or without the speaker’s face, four experimental lists were created, in which materials were rotated between conditions in a Latin square design. Participants were randomly assigned to one of these lists.

### Statistical analyses

The statistical analyses were performed using the statistical software R (R Core Team, 2023). The complete dataset and analyses scripts can be found in the OSF repository (https://osf.io/n42x9/). First, a preliminary accuracy check was performed, demonstrating that all participants achieved an accuracy level above 80% in both the lexical decision and comprehension questions. Only responses to words were considered (see Online Supplementary Materials for non-word data). Response accuracy was analyzed using generalized linear mixed-effects models (binomial distribution with logit link). Response times (RTs) of correct responses were log-transformed and analyzed using linear mixed-effects models (Gaussian distribution). All models were fitted with the lme4 package (Bates et al., 2015). Responses faster than 150 ms and slower than 2,500 ms were excluded from the analyses (0.007% of total observations). To find the best-fitting model for our data, we used a hierarchical model comparison approach (Heinze et al., 2018). The model comparison was based on the Akaike Information Criterion (AIC) and especially delta AIC and AIC weight as indexes of the goodness of fit. The AIC and AIC weight gives information on the models’ relative evidence (i.e., likelihood and parsimony), therefore the model with the lowest AIC and the highest AIC weight is to be preferred (Wagenmakers & Farrell, 2004). For both accuracy and RTs, model comparison included a null model with Participant and Item as random intercepts to account for participant-specific variability and item-specific idiosyncrasies (Baayen et al., 2008). Random slopes were not included due to the failure of tested models to converge. Predictor’s order was established giving priority to the main effects over interactions and to the effects (i.e., Accent and Constraint) with substantial support in the literature (Faust & Kravetz, 1998; Federmeier et al., 2007; Floccia et al., 2006, 2009; Munro & Derwing, 1995). Face and the related interaction terms were added subsequently in order to determine to what extent this variable increased the fitting of the model. Thus, inclusion of predictors followed this order: (i) Accent (Native vs. Foreign); (ii) Constraint (HC vs. LC); (iii) Face (Face vs. No Face); (iv) The two-way interaction between Constraint*Accent; (v) The two-way interaction between Constraint*Face; (vi) The three-way interaction between Constraint*Accent*Face.

Outliers were identified using the outlierTest function of the car package (Fox & Weisberg, 2019) and removed (0.0006 % of model observations). Sum coding was used as contrast coding in order to estimate main effects (Brehm & Alday, 2022). Post hoc comparisons were performed using the contrast function of the emmeans package (Lenth et al., 2023). P-values were adjusted using Bonferroni correction (Bonferroni, 1936).

## Results

### Accuracy

Descriptive statistics for accuracy according to conditions are reported in Table 1.

**Table 1:** Mean accuracy and standard deviation for each experimental condition

Accent	High Constraint		Low Constraint	
	No Face	Face	No Face	Face
Native	1 ± 0.01	1 ± 0.01	0.98 ± 0.04	0.98 ± 0.03
Foreign	0.95 ± 0.07	0.97 ± 0.04	0.79 ± 0.11	0.82 ± 0.10

As shown in Table 2, model comparison indicates that the best-fitting model (lower delta AIC and higher AIC weight) for accuracy is Model 3:

$$Accuracy \sim Accent+Constraint+Face+\left(1\left|Participant\right.\right)+\left(1\left|Item\right.\right)$$

**Table 2:** The comparison of GLMER models predicting accuracy

Models	Deviance	dAIC	AICw
M0. Accuracy ~ (1|Participant) + (1|Item)	2718.208	465.31	0.0
M1. Accuracy ~ Accent + (1|Participant) + (1|Item)	2291.899	41.0	0.0
M2. Accuracy ~ Accent + Constraint + (1|Participant)+ (1|Item)	2251.291	2.40	0.15
**M3. Accuracy ~ Accent + Constraint + Face + (1|Participant) + (1|Item)**	**2246.896**	**0.0**	**0.49**
M4. Accuracy ~ Accent + Constraint + Face + Constraint*Accent + (1|Participant) + (1|Item)	2246.482	1.59	0.22
M5. Accuracy ~ Accent + Constraint + Face + Constraint*Accent + Constraint*Face + (1|Participant) + (1|Item)	2245.961	3.07	0.11
M6. Accuracy ~ Accent + Constraint + Face + Constraint*Accent + Constraint*Face + Constraint*Accent*Face + (1|Participant) + (1|Item)	2244.698	5.80	0.03

Deviance = residual deviance; dAIC = difference between AIC of each model and the model with lower AIC; AICw = AIC weight

Model estimates for the best-fitting model for accuracy are reported in Table 3. The effect of Accent indicates that correct responses are less likely for foreign compared to native accent. The effect of Constraint indicates that correct responses are more likely in High-Constraint compared to Low-Constraint sentences. Finally, the effect of Face indicates that correct responses are more likely when the speaker’s face is present compared to when it is not present.

**Table 3:** Model estimates for the best-fitting model for accuracy

	Estimate	CI (95%)	Std. Error	z-value	*p*-value
Intercept	4.471	[4.061 4.881]	0.209	21.356	< .001
Accent: Foreign	-1.466	[-1.653 -1.279]	0.095	-15.363	< .001
Constraint: HC	1.012	[0.722 1.302]	0.148	6.841	< .001
Face: Face	0.124	[0.008 0.240]	0.059	2.092	.036

### Response times

Descriptive statistics for response times (ms) according to conditions are reported in Table 4.

**Table 4:** Mean response times (RTs) and standard deviations for each experimental condition

Accent	High Constraint		Low Constraint	
	No Face	Face	No Face	Face
Native	808.37 ± 220.42	767.20 ± 238.47	963.81 ± 213.86	958.62 ± 221.74
Foreign	980.90 ± 238.40	933.94 ± 245.21	1266.67 ± 291.45	1258.43 ± 297.76

As shown in Table 5, model comparison indicates that the best fitting model (lower delta AIC and higher AIC weight) for response times is Model 5:

$$LogRTS \sim Accent+Constraint+Face+Constraint\times Accent+Constraint\times Face+\left(1\left|Participant\right.\right)+\left(1\left|Item\right.\right)$$

**Table 5:** The comparison of LMER models predicting response times

Models	Deviance	dAIC	AICw
M0. LogRTs ~ (1|Participant) + (1|Item)	-519.555	2322.63	0.0
M1. LogRTs ~ Accent + (1|Participant) + (1|Item)	-2563.356	280.83	0.0
M2. LogRTs ~ Accent + Constraint + (1|Participant)+ (1|Item)	-2717.974	128.21	0.0
M3. LogRTs ~ Accent + Constraint + Face + (1|Participant) + (1|Item)	2767.483	80.70	0.0
M4. LogRTs ~ Accent + Constraint + Face + Constraint*Accent + (1|Participant) + (1|Item)	-2822.234	27.95	0.0
M5. LogRTs ~ Accent + Constraint + Face + Constraint*Accent + Constraint*Face + (1|Participant) + (1|Item)	**-2852.183**	**0.0**	**0.85**
M6. LogRTs ~ Accent + Constraint + Face + Constraint*Accent + Constraint*Face + Constraint*Accent*Face + (1|Participant) + (1|Item)	-2852.650	3.53	0.15

Deviance = residual deviance; dAIC = difference between AIC of each model and the model with lower AIC; AICw = AIC weight

**Fig. 2:**
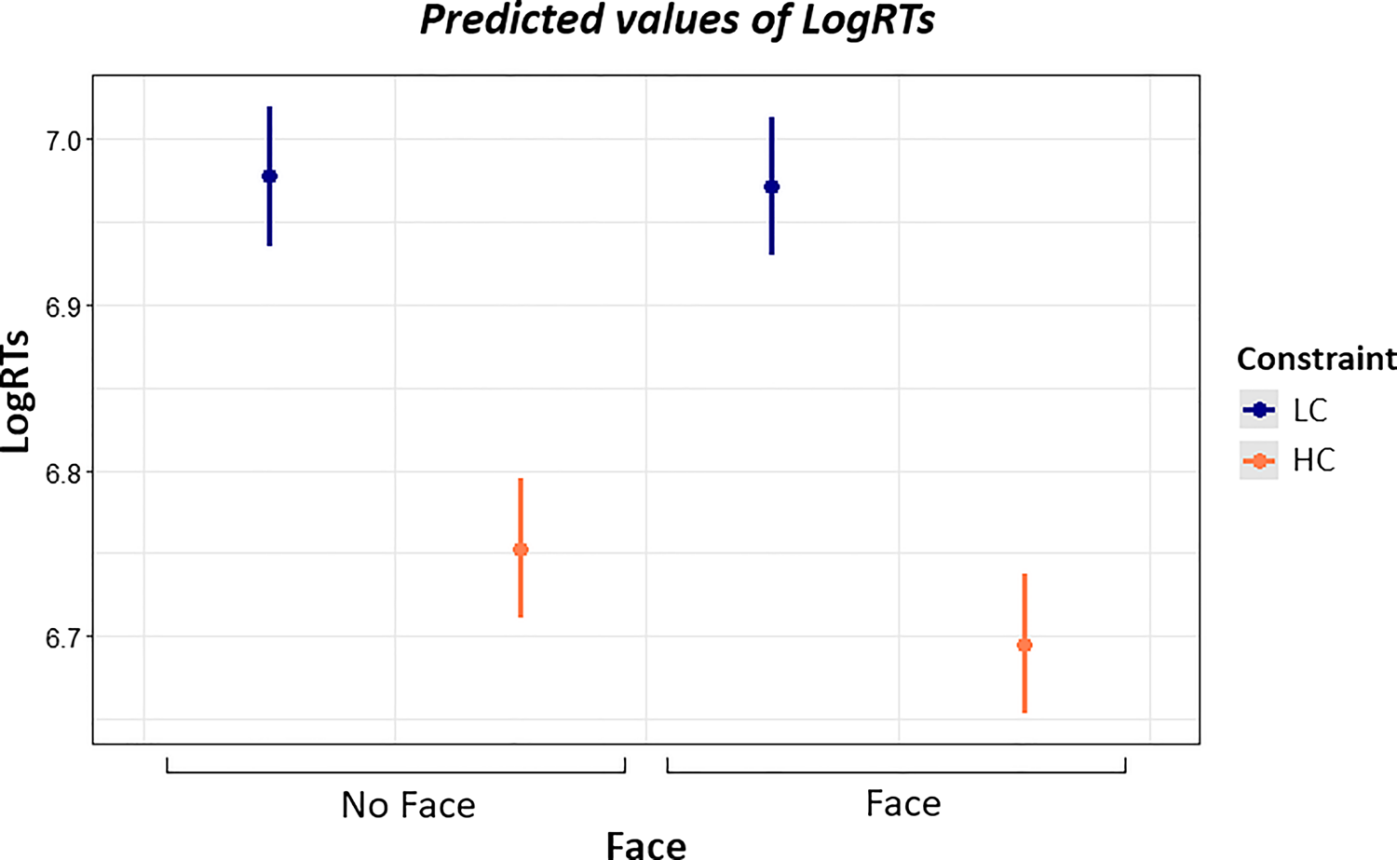
Model estimates for the interaction between Constraint*Face. The error bars indicate 95% confidence intervals

Model estimates for the best-fitting model for response times are reported in Table 6. The interaction between Constraint*Accent indicates that the Constraint effect, namely faster RTs for HC sentences compared to LC sentences, is larger for the foreign compared to the native accent. Importantly, the interaction between Constraint*Face indicates that cueing the speaker’s face is associated with an increased Constraint effect (Fig. 2).

**Table 6:** Model estimates for the best fitting model for LogRTs

	Estimate	CI (95%)	Std. error	t-value	*p*-value
Intercept	6.856	[6.817 6.896]	0.020	343.904	< .001
Accent: Foreign	0.120	[0.115 0.125]	0.002	51.004	< .001
Constraint: HC	-0.126	[-0.140 -0.112]	0.007	-17.533	< .001
Face: Face	-0.016	[-0.021 -0.011]	0.002	-6.864	< .001
Constraint*Accent	-0.017	[-0.022 -0.013]	0.002	-7.427	< .001
Constraint*Face	-0.013	[-0.017 -0.008]	0.002	-5.522	< .001

Post hoc comparisons have shown that cueing the speaker’s face is associated with faster RTs for HC sentences (*p* < .001) but not for LC sentences (*p* = .709). We found no evidence that this effect is modulated by the speaker’s accent (Native or Foreign) since the three-way interaction between Constraint*Accent*Face did not improve the model fit.

## Discussion

In the present study, we aimed to investigate the role of context information in predicting upcoming words, focusing on whether predictions occur at the phonological level of representation. To do so, we took advantage of the fact that foreign speakers often exhibit phonological errors and examined whether the prediction system is flexible enough to account for the phonological variability between speakers. In our experimental paradigm, the speaker’s face provides a cue to the phonological properties of the upcoming word before its presentation. The results showed that cueing the speaker’s face speeded up RTs only when the word was predictable. This facilitation seems to occur regardless of whether the face cued a foreign or a native accent. These results provide compelling evidence supporting the involvement of phonological representations in prediction and suggest that predictions rely on flexible and finely tuned processes capable of accommodating interindividual phonological variability. Previous experiments have shown that the prediction system is able to adapt to the specific speaker. For example, the extent to which comprehenders rely on predictive processing appears to be influenced by the reliability of the speaker (Brothers et al., 2017, 2019). Our findings extend this flexibility to predictions encompassing not only the semantic content of the speech but also the phonological form of the words.

Previous evidence indicated that phonological predictions were present when the sentential context was produced by a native speaker, but not when it was produced by a foreign speaker (Brunellière & Soto-Faraco, 2013). This pattern was attributed to less precise priors for prediction or a lower frequency of occurrence of the phonological variants in the mental lexicon when dealing with unfamiliar phonological contexts (Connine et al., 2008). However, our experiment showed that cueing the speaker's accent facilitated the recognition of words regardless of whether the accent was native or foreign. A possible explanation for these seemingly conflicting results could be due to the greater cognitive load associated with the processing of the sentential context when pronounced by a non-native speaker (Adank et al., 2009; Cristia et al., 2012; Floccia et al., 2006, 2009; Porretta et al., 2016, 2020). It has been shown that the availability of cognitive resources impacts predictive processes (Ding et al., 2023; Huettig & Mani, 2016; Otten & Van Berkum, 2009), and this occurs even in the comprehension of the second language (Ito et al., 2018). Thus, the reduction of phonological prediction found by Brunellière and Soto-Faraco (2013) is not incompatible with our results, but it just reveals a very different aspect of the interplay between predictive processing and non-standard speech. When the system does not have to deal with the uncertainty associated with the decoding of the speech of a non-native speaker very precise phonological predictions of the forthcoming speech can be made.

Our data also shed light on the processes involved in generating predictions. Cueing the speaker's face resulted in faster RTs only for predictable words, suggesting that this effect cannot be solely attributed to the *priming* of talker-specific representations (Creel et al., 2008; Creel & Bregman, 2011; Goldinger, 1996; Nygaard & Pisoni, 1998; Palmeri et al., 1993; Remez et al., 1997). Rather, our result appears to be specific to prediction processes based on sentential constraints. In this case, predictions seem to be generated through mechanisms that exploit all available linguistic and extralinguistic elements to anticipate the input, resulting in predictions that are tailored to the speaker. This result can hardly be explained by the spreading of activation among phonological abstract representations stored in long-term memory. The foreign speaker in our study produced words with phonological errors, and a pre-existing lexical representation of such phonological forms (namely lexemes) should not be available or at least it should be much less available compared to the phonological form of words spoken with standard phonology. Prediction-by-production accounts offer a valuable framework for understanding how listeners actively use the information about the speaker's phonological categories to generate predictions. According to these models, prediction during comprehension is supported by language production representations and mechanisms (Huettig, 2015; Pickering & Gambi, 2018; Pickering & Garrod, 2007, 2013), allowing listeners to generate predictions at different levels of representation, including the phonological level. Neurophysiology experiments using a paradigm similar to the one implemented here (Gastaldon et al., 2020, 2023; Piai et al., 2015), in which high- and low-constraining sentence frames were followed by a picture to be produced or by a target word to be perceived, showed very similar brain activations between production and comprehension. These results suggest that predicting the last word of high constraint sentences is at least partially subserved by the language production network. Although the behavioral paradigm implemented here does not explicitly support a prediction-by-production framework, our data are clearly compatible with this view. Covert imitation, a mechanism often emphasized by prediction-by-production models (Pickering & Gambi, 2018; Pickering & Garrod, 2013), could explain the flexibility of the prediction system. According to Pickering and Gambi (2018), covert imitation allows the transformation of comprehension representations into production representations. This mechanism could allow the system to generate predictions constrained by both the preceding sentential context and the speaker's accent.

The notion that covert imitation may adapt to the speaker’s accent aligns with findings demonstrating a direct relationship between overt speech imitation and speech comprehension. For instance, Adank et al. (2010) showed that participants who were instructed to overtly imitate a foreign accent demonstrated improved comprehension of foreign-accented sentences from background noise. Moreover, research in the field of speech perception has investigated the mechanisms by which listeners adapt to different speakers (Bradlow & Bent, 2008; Clarke & Garrett, 2004; Clopper & Bradlow, 2008; Maye et al., 2008; Weatherholtz & Jaeger, 2016). The system implicitly tracks and learns speaker-specific properties to optimally process the variations present in the environment and help listeners cope with talker variability (Kleinschmidt & Jaeger, 2015). For example, Nygaard et al. (1994) found that new words were recognized more accurately when produced by familiar speakers compared to new speakers. From this perspective, speech perception can be influenced by expectations about the speaker, such as their dialect background (Hay, Nolan et al., 2006a; Niedzielski, 1999), ethnicity (Casasanto, 2008), age (Drager, 2011; Hay, Warren et al., 2006b; Walker & Hay, 2011), and socio-economic status (Hay, Warren et al., 2006b). In our study, we propose that the perceptual adaptation to the speaker also extends to the predictive processes based on sentential constraint, possibly relying on production mechanisms.

Pickering and Garrod (2013) hypothesized that comprehenders prioritize prediction when they can predict accurately as in the case in which they can identify with the speaker. Therefore, in our experiment participants were expected to engage more in phonological prediction for the native speaker than for the foreign speaker. The results did not corroborate this expectation. A possible explanation might be associated with the systematic nature of our manipulation. The foreign-accented speech entailed altering three phonemes, and all target words began with one of these phonemes. Participants might have accurately anticipated the target phonology due to this structured pattern. Moreover, despite it is known that people tend to identify more with an in-group member, our paradigm was not aimed at manipulating this variable (likelihood of identification with the speaker) and thus it is a clearly suboptimal way to test this specific hypothesis of the Pickering and Garrod (2013) proposal.

The literature includes other prediction accounts proposing that comprehenders can actively predict upcoming speech without necessarily involving the production system. For instance, Kuperberg and Jaeger introduced a multi-representational hierarchical generative model (Kuperberg, 2016; Kuperberg & Jaeger, 2016) in which comprehenders rely upon internal generative models – a set of hierarchically organized internal representations - to probabilistically pre-activate information at multiple levels of representation. This pre-activation maximizes the probability of accurately recognizing the incoming information. Internal representations are built using both linguistic and non-linguistic information, and they may also include knowledge of the speaker’s sound structure (Connine et al., 1991; Szostak & Pitt, 2013). Listeners may learn different generative models corresponding to different statistical environments (Kleinschmidt & Jaeger, 2015), enabling them to consider the phonological variability between speakers when predicting upcoming words.

To conclude, comprehenders not only exhibit rapid adaptation to non-native speakers but also exploit the flexibility of the perceptual system to predict the upcoming speech even when it contains phonological errors. This provides valuable insights into both the level(s) of representation and the processes involved in generating predictions. Our results strongly support the notion that linguistic prediction involves the pre-activation of phonological representations, clearly showing that linguistic prediction processes go beyond the mere spreading of activation between long-term stored representations. Further research using not only a behavioral methodology (e.g., neurophysiology, neurostimulation or patient study) is warranted to gain a deeper understanding of the mechanisms involved in generating predictions at a sub-lexical level of representation and to determine the relative weight of these mechanisms. It would be also crucial to investigate the extent to which the effects found in the present study generalize to more ecological conversational settings, where participants engage in listening to contextual sentences. Finally, to better define what are the boundary conditions of the speaker effect in phonological predictions, it would be necessary to further develop paradigms aimed at controlling/manipulating the cognitive load requirements.

## Supplementary Information

Below is the link to the electronic supplementary material.

Supplementary file1 (DOCX 139 KB)

## Data Availability

The complete dataset and materials (including experimental materials and Supplementary materials) can be found in the Open Science Framework (OSF) repository (https://osf.io/n42x9/).

## Code availability

The analyses script can be found in the OSF repository (https://osf.io/n42x9/).
