# Supplementary material for: I know how you’ll say it: evidence of speaker-specific speech prediction
Source: Psychon Bull Rev. 2024 Mar 25;31(5):2332–44. doi: 10.3758/s13423-024-02488-2 (PMC11543741; doi:10.3758/s13423-024-02488-2)
Supplement: Supplementary file 1 — Supplementary file1 (DOCX 139 KB) [file 13423_2024_2488_MOESM1_ESM.docx]

**Supplementary materials**

We report the results for non-word rejection. Statistical analyses were performed using the same procedure as for words (see Statistical analyses section in the manuscript).

*Accuracy*
Descriptive statistics for accuracy in non-word rejection are reported in Table 1.

**Table 1**

*Mean accuracy and standard deviation for each experimental condition***.**

| ***Accent*** | ***High Constraint*** | | ***Low Constraint*** | |
| --- | --- | --- | --- | --- |
|  | ***No Face*** | ***Face*** | ***No Face*** | ***Face*** |
| ***Native*** | 0.99 ± 0.02 | 0.99 ± 0.02 | 0.99 ±0 .05 | 0.99 ± 0.04 |
| ***Foreign*** | 0.99 ± 0.02 | 0.99 ± 0.02 | 0.98 ± 0.03 | 0.99 ± 0.03 |

Non-word rejection was very accurate in all conditions. As a matter of fact, we used easily recognizable non-words. The very high accuracy of non-word rejection prevented us from performing statistical analyses on these data.

*Response times*

Descriptive statistics for response times (ms) in non-word rejection are reported in Table 2.

**Table 2**

*Mean RTs and standard deviation for each experimental condition.*

| ***Accent*** | ***High Constraint*** | | ***Low Constraint*** | |  |
| --- | --- | --- | --- | --- | --- |
|  | ***No Face*** | ***Face*** | ***No Face*** | ***Face*** | |
| ***Native*** | 1021.41 ± 254.68 | 979.41 ± 258.53 | 1113.02 ± 264.95 | 1086.27 ± 265.76 | |
| ***Foreign*** | 1174.51 ± 345.75 | 1169.28 ± 342.99 | 1298.09 ± 357.65 | 1325.13 ± 372.62 | |

As shown in Table 3, model comparison indicates that the best fitting model for response times is Model 6: *LogRTs ~ Accent + Constraint + Face + Constraint*Accent + Constraint*Face + Constraint*Accent*Face + (1|Participant) + (1|Item).***Table 3**

*The comparison of LMER models predicting response times.*

| **Models** | **Deviance** | **dAIC** | **AICw** |
| --- | --- | --- | --- |
| M0. LogRTs ~ (1\|Participant) + (1\|Item) | -1858.388 | 1233.87 | 0 |
| M1. LogRTs ~ Accent + (1\|Participant) + (1\|Item) | -3024.513 | 69.75 | 0 |
| M2. LogRTs ~ Accent + Constraint + (1\|Participant) + (1\|Item) | -3064.793 | 31.47 | 0 |
| M3. LogRTs ~ Accent + Constraint + Face + (1\|Participant)  + (1\|Item) | -3074.070 | 24.19 | 0 |
| M4. LogRTs ~ Accent + Constraint + Face + Constraint*Accent  + (1\|Participant) + (1\|Item) | -3079.867 | 20.39 | 0 |
| M5. LogRTs ~ Accent + Constraint + Face + Constraint*Accent  + Constraint*Face + (1\|Participant) + (1\|Item) | -3086.350 | 15.91 | 0 |
| **M6. LogRTs ~ Accent + Constraint + Face + Constraint*Accent  + Constraint*Face + Constraint*Accent*Face + (1\|Participant)  + (1\|Item)** | **-3106.260** | **0.0** | **1** |

**Note.** Deviance = residual deviance; dAIC = difference between AIC of each model and the model with lower AIC; AICw = AIC weight

Model estimates for the best-fitting model for response times are reported in Table 4. The three-way interaction between Accent*Constraint*Face is statistically significant (*p* < .01 for the contrasts reported in LMER output*; p* < .001, for the model term tested with the “anova” function).

**Table 4**

*Model estimates for the best fitting model for LogRTs.*

|  | ***Estimate*** | ***CI (95%)*** | ***Std. Error*** | ***t-value*** | ***p-value*** |
| --- | --- | --- | --- | --- | --- |
| *Intercept* | 7.010 | [6.9654 7.0538] | 0.022 | 315.244 | < .001 |
| *Accent: Foreign* | 0.0816 | [0.0772 0.0860] | 0.002 | 36.154 | < .001 |
| *Constraint: HC* | -0.0559 | [-0.0719 -0.0400] | 0.008 | -6.915 | < .001 |
| *Face: Face* | -0.0066 | [-0.0110 -0.0022] | 0.002 | -2.931 | .003 |
| *Accent[Foreign]*Constraint [HC]* | -0.0053 | [-0.0097 -0.0009] | 0.002 | -2.353 | .019 |
| *Constraint[HC]*Face [Face]* | -0.0057 | [-0.0102 -0.0013] | 0.002 | -2.538 | .011 |
| *Accent[Foreign]*Constraint [HC]*Face[Face]* | 0.0104 | [0.0042 0.0166] | 0.003 | 3.264 | .001 |
| *Accent[Foreign]*Constraint [LC]*Face[Face]* | 0.0102 | [0.0039 0.0165] | 0.003 | 3.165 | .002 |

Figure 1 illustrates the three-way interaction between Accent*Constraint*Face. Cueing the speaker’s face seems to be associated with faster RTs in the Native accent condition. Post-hoc comparisons showned that cueing the native speaker’s face is associated with faster RTs in both HC (*p* < .001) and LC sentences (*p* = 0.027). In the Foreign accent condition cueing the speaker’s face seems to be associated with slower RTs in LC sentences. However, post-hoc comparisons have shown no evidence that cueing the foreign speaker’s face influences RTs in both HC (*p* = 1) and LC sentences (*p* = 0.086).

**Figure 1**

*Model estimates for the interaction between Accent*Constraint*Face. The error bars indicate 95% confidence intervals.*


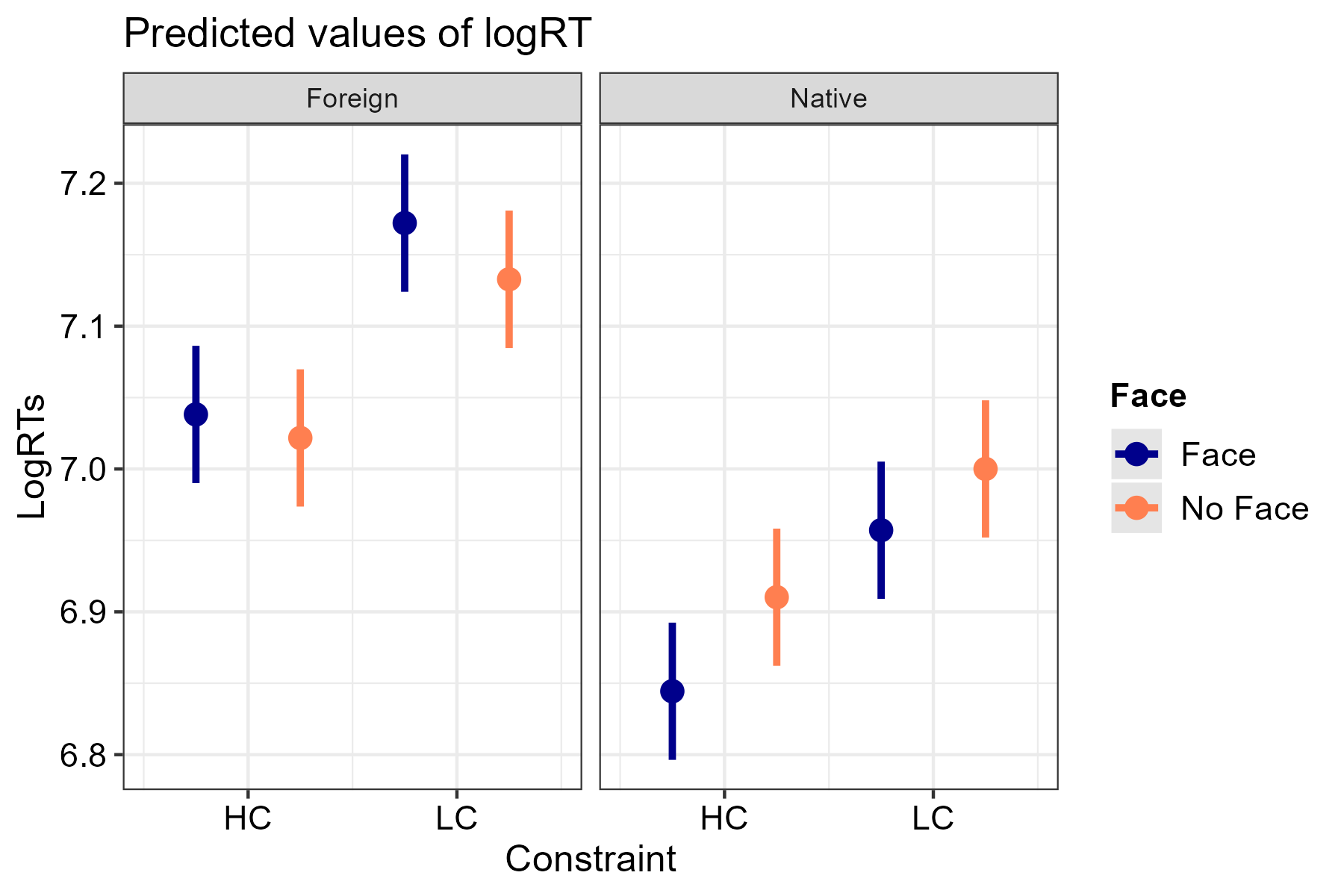


In the rejection non-words, the face effect was observed in the native accent but not in the foreign accent condition. This pattern might be due to the fact that, when the face of the native speaker was cued, participants could reject non-words uniquely on the basis of the phonological form of the stimuli. This strategy could not be used when the face of the foreign speaker was cued, since foreign accent words always contained phonological errors.
